# Utilizing Listening Sessions to Assess COVID-19 Vaccine Acceptance among Asian Americans in Michigan

**DOI:** 10.3390/healthcare10112284

**Published:** 2022-11-14

**Authors:** Olivia Ford, Rachel Bessire, Alice Jo Rainville, Tsu-Yin Wu

**Affiliations:** 1 Dietetics and Human Nutrition, Eastern Michigan University, Ypsilanti, MI 48197, USA; 2 Center for Health Disparities Innovations and Studies, Eastern Michigan University, Ypsilanti, MI 48197, USA

**Keywords:** vaccine acceptance, COVID-19, Asian American, community health, listening sessions

## Abstract

SARS-CoV-2 (COVID-19) hospitalizations and deaths have been in the forefront of healthcare and public health for the past two years. Despite widespread vaccinations campaigns, infection rates and serious illness and death remain high among immigrant and minority communities. There are many factors that increase the risk of hospitalization and death, including overall health of the individual as well as environmental and socioeconomic factors. Seven virtual listening sessions with 39 Asian American adults were conducted to assess acceptance of COVID-19 vaccines. Lack of access, confusion on eligibility, distrust of mass vaccination sites, and fear of long-term side effects were primary barriers to vaccine acceptance. Perspectives on the vaccines varied by ethnic groups, with Bangladeshi and Yemeni participants more likely to have negative views. Our findings show that while national statistics of the broad category “Asian” indicate higher COVID-19 vaccination rates than other minority groups, there are Asian ethnic groups that may not follow these trends. These groups are important to prioritize as they may be at increased risk for exposure and severe illness. However, these groups can be difficult to access for reasons such as language barriers and cultural norms. Information from these listening sessions was used to create resources and programs to clarify misconceptions and increase access to COVID-19 vaccines.

## 1. Introduction

The burden of SARS-CoV-2 (COVID-19) hospitalizations and deaths has been in the forefront of healthcare and public health for the past two years. According to the Centers for Disease Control and Prevention, as of 14 May 2022, there were over 1 million deaths in the U.S. and over 32,000 deaths in Michigan involving COVID-19 [1]. The COVID-19 pandemic has additionally been costly in terms of healthcare dollars with medical costs for hospitals in Michigan ranging from an average charge of $35,082 for a noncomplex COVID-19 patient to $207,926 for a complex COVID-19 patient who requires ventilation and/or admission to the intensive care unit [2]. There are many factors that increase the risk of hospitalization and death, including overall health of the individual as well as environmental and socioeconomic factors. Bajgain et al. [3] conducted a systematic review of 27 studies and found that hypertension, cardiovascular disease, and diabetes were the most common comorbidities found in patients who died due to COVID-19. An estimated 84.1% of these patients had one or more comorbidities. Although risk varies by ethnic group, Asian Americans are at increased risk for several of the comorbidities associated with death from COVID-19 [4].

The COVID-19 pandemic has had a greater impact on immigrants and minorities; they have experienced higher infection rates due to factors such as increased housing density, employment as essential workers, lack of access to healthcare, diminished quality of care, and many experience a combination of these factors [5]. In addition to being at higher risk for infection, immigrants and minorities such as Asian Americans are at increased risk for serious illness and death, in part due to underlying health conditions.

Despite increased risk of infection and death, minority populations typically have lower vaccine uptake with the exception of Asian Americans [6]. As of 10 January 2022, the overall COVID-19 vaccination rate for Asian Americans was higher compared to Whites (81% vs. 60%) across the U.S. as well as African Americans (54%) and Hispanics (60%) [6]. COVID-19 vaccines were not the only instance where Asian Americans had higher rates of vaccination. For example, flu vaccination coverage in the 2020–2021 flu season was 54.5% among Asian American adults, 40.4% among non-Hispanic Black adults, 38.6% among Hispanic or Latino adults, 41.5% among American Indian or Alaska Native adults, and 55.5% among non-Hispanic White adults [7]. However, some Asian Americans, particularly those with lower income, may face barriers for vaccination, such as limited English proficiency and internet access. For example, in Hamtramck, Michigan, one of our areas for research, 82.9% have a computer and 66.8% have a broadband internet subscription, whereas overall in Michigan, 91.1% of households have a computer and 84.4% have a broadband internet subscription [8]. Access to a computer and the internet is important to consider as this was the only way to register for a COVID-19 vaccination appointment at the outset of the vaccine roll-out and continues to be a primary means for the dissemination of information and appointment registration. Michaels et al. (2021) [9] described internet access as a social determinant of health, and their research found a significant association between lack of internet access and low COVID-19 vaccination rates in New York City zip codes.

There are many factors that affect vaccine acceptance, and these can vary between race and ethnic groups as well as genders. The type and source of information can play an influential role as well. Kricorian and Turner [10] found that African Americans and Latinos were more likely to take a vaccine if it was supported by a medical professional of their race or ethnicity. Findings of Jacobi and Vaidyanathan [11] also support the notion that individuals of race or ethnic minority are more likely to trust information that came from their own community. Gorman et al. [12] found that study participants saw physicians and primary care providers to be their most trusted source of information. Gender differences were also found as women were more likely than men to be vaccine hesitant [11]. Vaccine acceptance may not be affected by the level of knowledge about COVID-19 vaccines. Kumari and colleagues [13] conducted eight focus groups in India and found the level of knowledge about COVID-19 vaccines (e.g., awareness of COVID-19 vaccines, names of vaccines rolled out in India, and number of vaccine shots to be administered) did not correlate with a positive attitude towards COVID-19 vaccines as both hospital-based groups and the general population had mixed views. While existing studies have provided data on vaccine perceptions among African Americans, Latinos, and Asians, the work presented here bridges a gap in the literature by examining Asian Americans’ perceptions of COVID-19 vaccines.

In this study, seven listening sessions (similar to focus group discussions) were held between April 2021 and January 2022 for six Asian ethnic groups that are widely represented in Southeast Michigan: Bangladeshi (two sessions), Burmese, Filipino, Korean, Arab (Yemeni), and Japanese. The purpose of these listening sessions was to elucidate how COVID-19 impacted communities and how participants and their communities viewed COVID-19 vaccines. Information from these listening sessions was used to create resources and programs to clarify misconceptions and increase access to COVID-19 vaccines. A virtual listening session format was chosen for our research because it increased anonymity for participants, allowed them to answer questions in the security and comfort of their own home, was safer than meeting in person, and required less time commitment from participants [14]. Dos Santos Marques et al. [15] found that virtual focus group participants (minority surgical patients) were more relaxed and engaged in virtual discussions. With respect to data collected, research has shown that in-person groups and online groups are nearly equivalent; while in-person groups can yield 260% more words than online groups, online groups are better at responding to the questions asked, more professional, and more efficient with their answers [16]. There are disadvantages to virtual listening sessions, which include the requirement for technology and access to internet service and a meeting platform, such as Zoom.

## 2. Materials and Methods

Prior to conducting the virtual listening sessions, Center for Health Disparities Innovations and Studies (CHDIS) research team members developed a script and a 13-item survey based on the CDC’s COVID-19 Vaccine Confidence Rapid Community Assessment Guide [17]. The consent form and 13-item survey were completed in Google Forms prior to the start of the listening sessions. Data collected from the surveys are presented in Table 1 and Table 2. Only research team members were provided with access to the Google Forms. The survey was available in English.

Community-based partners assisted in recruiting 4–9 participants for each virtual listening session. This group size was estimated to be an appropriate size that would lead to maximum engagement by all participants [18]. We over-recruited with the understanding that not all potential participants would attend the listening session. Groups were determined by race/ethnicity and in the case of Bangladeshi participants, by gender as well (Table 1). Listening sessions were conducted via Zoom and lasted approximately one hour. The listening sessions were held in English with translators available. Sessions were video and audio recorded and transcribed into Word. The listening session script is provided in Appendix A. Participants were notified that they would be recorded and that all personal identifiers would be removed from the transcript. They were allowed to turn off their cameras if they wished. After the listening sessions, the research team mailed $15 gift cards to the participants as tokens of appreciation. Two members of the research team analyzed the transcripts for themes, and quotes were categorized by theme. Data from the online surveys was entered into IBM^®^ SPSS^®^ software, and frequencies were analyzed.

All methods and procedures were approved by the Eastern Michigan University Institutional Review Board for Human Subjects.

## 3. Results

There were a total of 39 participants among the seven listening sessions. Races/ethnicities represented included Bangladeshi, Burmese, Filipino, Korean, Arab (Yemeni), and Japanese. The demographics of the listening session participants are shown in Table 1. The majority of the participants identified as female (74.4%), and most had completed some college education or had completed a bachelor’s degree or higher (74.4%). The mean age of the participants was 42.73 (SD = 20.04). The Filipino participants had the oldest mean age (59.87 years, SD = 15.14), while the Arab participants had the youngest mean age (21.33 years, SD = 1.63).

Over half of the participants (51.3%) reported that they spoke English “very well” (Table 1). However, this varied by ethnicity, with 77.8% of Arab (Yemeni) participants reporting that they spoke English “very well” but no Burmese or Filipino participants rating themselves as such. The most common health condition reported was type 2 diabetes (7.7%). Of the participants, 59% had ever been tested for COVID-19, and 84.6% reported not ever having COVID-19 (Table 2).

### 3.1. Themes from Listening Session

#### 3.1.1. Perceptions of COVID-19 Vaccine Benefits

Overall, listening session participants expressed positive views about COVID-19 vaccines (Table 3). Gratitude for COVID-19 vaccines and a sense of increased safety were expressed by most groups. “It’s really, really good to have the vaccine, because after we got the vaccine we feel safe, and now we can go anywhere” (Burmese). Participants felt comfortable engaging in more activities and interacting with a greater number of people after being vaccinated. “…now I’m okay to go outside and walk and talk to a lot of people even just you know wearing a mask and I feel confident” (Filipino). Some of the groups, including Burmese, Filipino, and Korean, indicated that most of their community members had been vaccinated.

Participants from other communities indicated there was a sense of division surrounding COVID-19 vaccination status. A Bangladeshi woman noted, “even amongst my own family members is it’s very divided. It’s either ‘I’m not getting a vaccine’ or ‘I am getting a vaccine,’ there’s no middle ground per se”. Some of this division was associated with age and therefore perceived risk. “It’s usually the older generation that’s concerned, like gen Z isn’t concerned, the millennials aren’t concerned but older generations are” (Yemeni). “You know, healthier, freer, younger people kind of hesitate and show that hesitation, I just wonder what kind of thinking is going through their mind” (Japanese). Several of the groups expressed that older generations were more likely to get vaccinated. “I see a lot of young people bringing their parents and grandparents to get vaccinated, which is good”. For the Filipinos, vaccination is considered a way of life: “we’re used to receiving vaccines back in the Philippines from when we were younger”. However, one Filipino participant stated that the young people were less accepting of COVID-19 vaccines because “they feel that they are invincible”.

Some groups discussed community preferences with respect to the three available vaccines. Members of the Korean session expressed that the Johnson and Johnson (J&J) vaccine was preferred by the seniors because it was only one shot, while Bangladeshi women expressed that their community did not prefer the J&J vaccine because of the fears surrounding myocarditis. “Right after the Johnson & Johnson vaccine got shelved, people said, ‘if you already have heart conditions, or whatever you shouldn’t get this vaccine, you know you’ll die with stroke or another heart attack’ and how do you combat that”? (Bangladeshi).

#### 3.1.2. Locations of COVID-19 Vaccine Uptake

Participants shared their thoughts regarding the variety of locations (doctor’s office, pharmacy, mobile clinics, etc.) where vaccinations were offered, and this was a new concept for some. One Korean participant stated, “It’s very interesting to me because in my opinion, for South Korea, I think that the government, they organize all the vaccine schedule, but here in the US, there are a lot of source [s]”. Vaccine locations varied, with some receiving vaccines at a pharmacy, some at a mobile clinic sponsored by a local community organization, and others at their primary care physician’s office. While mass vaccination sites (e.g., Ford Field in Detroit) were instituted in the early phase of the vaccine roll-out to get large numbers of individuals vaccinated, participants shared negative feelings, such as fear, about these sites. “You know there there’s definitely a fear that, if you don’t have the right paperwork and you’re not legally here, then they don’t want to show up to get a vaccine. And, especially when you start hearing about the Ford Field and the Army administering the vaccine that’s the type of fear it imposes on people” (Bangladeshi). Additionally, mass vaccination sites can be difficult to navigate, particularly for individuals who are not proficient in English, and there may be distrust of police and military presence.

#### 3.1.3. Misconceptions and Myths about COVID-19 Vaccine

Participants shared reasons they were hesitant to be vaccinated and/or concerns they heard from family, friends, and community members. Several of these reasons/concerns were recurring. One example was the fear of adverse side effects. “I know some Japanese person, Japanese woman, who refused to get [a] vaccine because she believes [the] COVID vaccine will destroy the immune system and if you get [the] vaccine you will get sick easily” (Japanese). It was mentioned in all sessions that some community members were afraid of getting the vaccine because of the fear of long-term effects. “But the community that I’m around they’re afraid of taking the vaccine because they don’t know the long-term effects of the vaccine and they don’t know if it would harm them 40 years from now, 20 years from now” (Yemeni). Additionally, some of the female participants discussed concern about the impact of vaccinations on their fertility.

In addition to fear related to side effects, participants discussed fear, whether their own or of a family member/friend, that the vaccines were developed too quickly to be safe. “They don’t really understand the science of it and its new” (Bangladeshi). One Yemeni participant noted that the vaccine had “bad ingredients”, and another said, “The vaccine did come out really fast and people are skeptical like, okay, why did this vaccine come out super-fast”? (Yemeni). Religious objections were varied and included sentiments that “God will protect me” in the Bangladeshi community to the theory that the vaccine is “a conspiracy about mind control” and “is meant to alter our DNA and replace it with a 666 sign” (Filipino). In the Burmese group, participants mentioned twice that some people do not want to get the vaccine if they have already had COVID-19 because they fear they will get sick again. Another concern was that incentives were offered to take the COVID-19 vaccine. “What really gets to me is that many companies or schools or healthcare places always try to bribe the people to take the vaccine with money to get it makes me feel worried” (Yemeni). The notion of being bribed left people feeling as though there must be an ulterior motive (e.g., mind control) behind government promotion of the vaccines.

Another source of fear was misinformation from young mothers’ online groups, Google, and distrust of medical professionals. In the listening session with primarily young Yemeni mothers, one participant said, “You hear a lot of moms all over the United States talking about how, when they vaccinated their child their bodies reacted differently to the vaccine and some moms lost their children because of the vaccines that they received” (Yemeni). Another participant expressed that they could not trust Google as a good source of information: “Google is so horrible. Google can make you happy or scare you to death, so Google I can never trust” (Yemeni). “Proper access to information is definitely an issue” (Bangladeshi). Others questioned the veracity of medical professionals: “It’s like they’re forced to say that and we’re like is it really true, is that what being a doctor all about? Telling us what we need to hear so they can get paid, or is it really like real research, like real truth that we need to hear” (Yemeni).

#### 3.1.4. Barriers to Vaccination

Aside from fear and misinformation as described in the section above, the primary barrier was access. This included shortage of appointments as well as difficulty utilizing the online scheduling system. “I remember, there was a whole lot of confusion in the beginning of who qualifies and where do I sign up, and how do I go” (Bangladeshi). “One was the making a reservation through [the] Internet, and that was very, very difficult. When they call in there’s not many people [to] answer or you have to wait for a long time” (Korean). However, these factors seemed to diminish as time progressed and vaccines became available to the general public. In one listening session conducted in October 2021, it was expressed that “people know where to go and get them for those that wanted to go” (Bangladeshi). Another nuance that affected vaccine access was city borders as Hamtramck is surrounded by Detroit and shares a border with Highland Park. A Bangladeshi participant noted, “telling Hamtramck residents, you can only get it at these places, and then telling Detroit Bangladeshi residents—actually you can’t get it in Hamtramck- you have to actually go further out to some other places” (Bangladeshi). This led to confusion because many Detroit Bangladeshi residents consider themselves part of the Hamtramck Bangladeshi community regardless of their physical home address.

In the Bangladeshi community, there is an added level of difficulty in reaching everyone because of their customs. “The Bangladeshi-led mosques are you know, usually people who are congregating there are the men in the community, so that information doesn’t trickle down to the women in the community, unless they have a male family member who is regularly attending”. Connecting with women’s groups was a suggested means for addressing this.

#### 3.1.5. Confusion about COVID-19 Vaccines

There was general consensus that the media was used to obtain information regarding the pandemic and COVID-19 vaccines but that many community members were utilizing unreliable sources, such as politically motivated news sources and social media influencers who lack the scientific/medical education needed to make qualified statements regarding COVID-19 and the associated vaccines. Participants presented mixed views on the media’s role in providing vaccine information. A Filipino participant said, “I think the media has helped a lot in the dealing with things”, while another stated, “CDC sometimes conflicts with what Fauci [is] saying. I think it all adds up to the reason why we are not achieving a better percentage of people being vaccinated”. A Bangladeshi participant noted that culturally specific media (i.e., a paid subscription is required to access culturally specific radio and/or television channels) that provided credible information was not free. Participants shared that local radio stations promoted vaccine hesitancy, while public health departments, hospitals, and local Asian American community organizations promoted reliable information about COVID-19 vaccines. This mixed messaging led to confusion about who to trust. “I don’t know, it’s like we don’t know who to believe anymore, we don’t know who’s telling the truth and who’s not” (Yemeni).

#### 3.1.6. Suggestions for COVID-19 Vaccine Messaging

Listening session participants were asked for suggestions on how to reach their community members with information and for suggestions on what information their community lacked. The notion of doing one’s part for the community even if one did not feel they were at risk themselves was discussed. Other suggested topics included information on how COVID-19 vaccines work, what side effects to expect, what percentage of people get side effects, and what medications can be taken (i.e., painkillers) to mitigate the common side effects of the vaccines. A variety of methods were suggested for reaching community members. The Bangladeshi participants suggested including a flyer with the local water bill as these are distributed to all households. Local group leaders, union leaders, faith leaders, and doctors were mentioned as trusted community members who could encourage people to get vaccinated. However, not all participants were willing to trust community members. “Honestly I just I feel like there’s not really not many people I would listen to in my community that would convince me, other than my doctors” (Yemeni).

One of the community partners noted great success with joint efforts in communication between their community organization and healthcare professionals, including doctors and community health workers, who know the language and cultures: “we’ve done multiple translations and videos surrounding this topic and the pandemic and we also partnered with our local hospital. We had a doctor from the hospital who came and talked to us about the COVID-19, the COVID-19 vaccine, and the importance” (Burmese). Having a well-known community center where they have multiple interpreters facilitated vaccinations for the Burmese community: “We just can just go there, so it’s really easy. and they had a fixed schedule already so it’s really helpful for all the Burmese” (Burmese). Effective strategies for promoting COVID-19 vaccination messaging include disseminating success stories, flyers and infographics translated in native Asian languages, and providing gift card incentives from community sources compared to government or business sources to earn trust from community residents.

## 4. Discussion

Although not directly asked, many participants shared their vaccination status. Of those who shared, more had been vaccinated or planned to be vaccinated than those who had not. Additionally, many expressed positive personal views of COVID-19 vaccines. However, there was variation in views amongst the listening session groups. Bangladeshi and Yemeni participants were more likely than other participants to not be vaccinated and to state that they did not plan to get vaccinated. There were some consistencies regarding limiting factors for vaccine uptake, which included language barriers and distrust of vaccine safety, which was fueled by misinformation. While there is limited data available on vaccine acceptance among Asian Americans, similar findings have been reported for other minority groups and Asians in other countries. Dong et al. [19] found that Black/African American and Hispanic/Latino adults were fearful of safety of COVID-19 vaccines and preferred to “wait and see” how the vaccines affected others. Siu et al. [20] reported that Chinese older adults living in Hong Kong expressed concerns about medical side effects of COVID-19 vaccines and felt they had been developed too quickly.

Some of the limiting factors dissipated over time. Lack of access, confusion on eligibility, and distrust of mass vaccination sites were discussed as limiting factors early in the vaccine roll-out, but vaccines were readily available and community members were able to receive vaccines from a trusted source when we conducted the listening sessions. The limiting factor that persisted was fear/distrust of vaccine safety. To address this, CHDIS collaborated with community-based organizations to host mobile COVID-19 vaccine clinics at locations community members are familiar with and trust (e.g., mosques and community centers). “They were able to come and get a vaccine because it’s from the trusted source of within either church or within community. We send out the information, they know us, and so they come to a trusted place for getting their vaccine” (Korean). The importance of trusting the source of information was also presented in the listening sessions. This is consistent with the findings of Gorman et al. [12] and Jacobi and Vaidyanathan [11], who reported that individuals of racial/ethnic minority communities are more likely to trust COVID-19 vaccine information that comes from their physician or trusted community member, respectively. In response, CDHIS team members trained 51 trusted messengers who were members of the community and partnered with local health professionals in an effort to provide consistent and accurate information regarding COVID-19 vaccines to community members.

This study presents data on a unique priority population: Asian Americans. In fact, we were not able to find any comparable research in the literature. Asian Americans are often deemed “model minorities” with respect to health and socioeconomic status and are therefore excluded from health-related research and not targeted with public health programming [21]. Our findings show that while national statistics of the broad category “Asian” indicate higher COVID-19 vaccination rates than other minority groups, there are Asian ethnic groups that may not follow these trends. These groups are important to target as they may be at increased risk for exposure and severe illness. However, these groups can be difficult to access for reasons such as language barriers and cultural norms.

**Figure 1:**
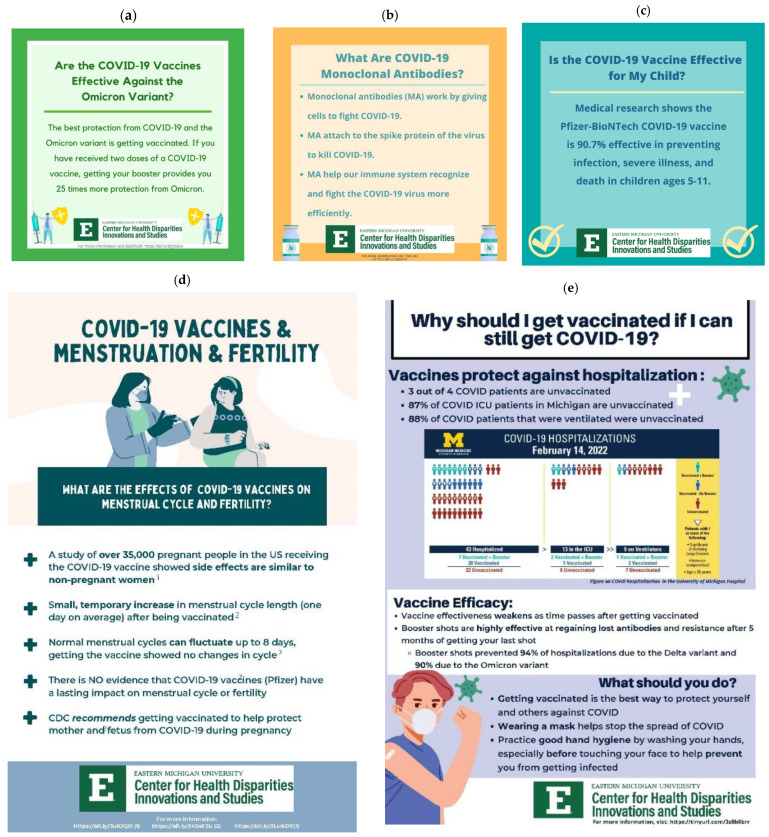
Examples of messaging/education materials created for priority population based on listening session feedback: (**a**) effectiveness against Omicron; (**b**) monoclonal antibodies; (**c**) vaccination effectiveness in children; (**d**) menstruation and fertility, (**e**) why get vaccinated if I can still get sick.

Findings of the listening sessions were used to create culturally relevant educational materials (see Figure 1) with the goals of dispelling misinformation and increasing COVID-19 vaccination uptake. These materials were translated into eight Asian languages and promoted through social media and local Asian community partner organizations. In addition, 41 mobile vaccine clinics were conducted in partnership with trusted community organizations, and 3731 vaccinations were given between March 2021 and May 2022. We also created a virtual listening session toolkit [22], which was shared by the CDC with other Racial and Ethnic Approaches to Community Health (REACH) recipients.

### Limitations

There are limitations to this study. The findings may not be generalizable to non-Asian races or ethnicities. There was variation in the number of participants in the listening sessions, so some Asian subgroups and communities had greater representation in the results than others (Table 1). Similarly, 74.4% of the participants identified as female, while 25.6% identified as male. Listening sessions were conducted via Zoom to limit the need for participants to travel and limit possible COVID-19 exposure. Therefore, individuals who did not have access to internet service and/or Zoom were not represented in our study findings. These individuals may have different barriers to access and different opinions on COVID-19 vaccines. Finally, language and health literacy barriers may have skewed study findings.

## 5. Conclusions

Overall, the listening session group participants were more likely to have a positive attitude towards the vaccines, similar to the findings by Bhuiyan et al. [23]. While not assessed formally, participants seemed knowledgeable about vaccines, but as previous research has shown, the level of knowledge may not be correlated with vaccine acceptance [13]. Therefore, addressing concerns about vaccine safety and removing access and language barriers may be instrumental tools in successful vaccination campaigns.

## Figures and Tables

**Table 1:** Demographics of Asian American COVID-19 listening session participants obtained from survey (Appendix A).

	All Participants	Bangladeshi	Burmese	Filipino	Korean	Arab (Yemeni)	Japanese
Sample size	39	7 (17.9%)	4 (10.3%)	8 (20.5%)	7 (17.9%)	9 (23.1%)	4 (10.3%)
Gender							
Male	10 (25.6%)	3 (42.9%)	0	4 (50.0%)	3 (42.9%)	0	0
Female	29 (74.4%)	4 (57.1%)	4 (100.0%)	4 (50.0%)	4 (57.1%)	9 (100.0%)	4 (100.0%)
Age (mean, SD)	42.73 (20.04)	33.57 (15.26)	40.00 (9.85)	59.87 (15.14)	49.4 (17.5)	21.33 (1.63)	50.25 (27.48)
Education level							
Less than high school	1 (2.6%)	1 (14.3%)					
High school	9 (23.1%)	1 (14.3%)	2 (50.0%)	1 (12.5%)	1 (14.3%)	5 (55.6%)	1 (25.0%)
Some college	12 (30.8%)	3 (42.9%)	2 (50.0%)		6 (85.7%)	4 (44.4%)	1 (25.0%)
Bachelor’s degree or higher	17 (43.6%)	2 (28.6%)		7 (87.5%)			2 (50.0%)
Employment status							
Not working—laid off	2 (5.1%)	1 (14.3%)					1 (25.0%)
Not working—retired	6 (15.4%)			3 (37.5%)	1 (14.3%)		2 (50.0%)
Not working—student	9 (23.1%)	2 (28.6%)			1 (14.3%)	5 (55.6%)	1 (25.0%)
Not working—leave of absence	2 (5.1%)		1 (25.0%)	1 (12.5%)			
Not working—temporarily laid off	1 (2.6%)					1 (11.1%)	
Stay-at-home mom	1 (2.6%)					1 (11.1%)	
Both remotely and in person	6 (15.4%)	2 (28.6%)	1 (25.0%)	2 (25.0%)			
In person only	6 (15.4%)	2 (28.6%)	2 (50.0%)	1 (12.5%)	1 (14.3%)	2 (22.2%)	
Remotely only	6 (15.4%)	1 (14.3%)		1 (12.5%)	4 (57.1%)		
English proficiency							
Very well	20 (51.3%)	5 (71.4%)		4 (50.0%)	3 (42.9%)	7 (77.8%)	1 (25.0%)
Well	14 (35.9%)	2 (28.6%)	1 (25.0%)	4 (50.0%)	3 (42.9%)	2 (22.2%)	2 (50.0%)
Not well	5 (12.8%)		3 (75.0%)		1 (14.3%)		1 (25.0%)
Primary spoken language							
Arabic	4					4 (44.4%)	
Bangla	4	4 (57.2%)					
Bosnian	1					1 (11.1%)	
Burmese	2		2 (50.0%)				
Chin/Falam	1		1 (50.0%)				
English	15	3 (42.9%)		5 (62.5%)	2 (28.6%)	4 (44.4%)	1 (25.0%)
Filipino	3			3 (37.5%)			
Japanese	3						3 (75.0%)
Korean	5				5 (71.4%)		
Tedim/Zomi	1		1 (25.0%)				

**Table 2:** Health conditions of COVID-19 listening session participants as reported via survey.

	All Participants	Bangladeshi	Burmese	Filipino	Korean	Arab (Yemeni)	Japanese
Sample size	39	7 (17.9%)	4 (10.3%)	8 (20.5%)	7 (17.9%)	9 (23.1%)	4 (10.3%)
Health conditions							
Cancer *	1 (2.6%)			1 (12.5%)			
Heart conditions	1 (2.6%)			1 (12.5%)			
Obesity	1 (2.6%)					1 (11.1%)	
Type 2 diabetes	3 (7.7%)	1 (14.3%)		1 (12.5%)	1 (14.3%)		
Difficulty doing errands alone							
Yes	1 (2.6%)				1 (14.3%)		
No	37 (94.9%)	7 (100%)	4 (100%)	7 (87.5%)	6 (85.7%)	9 (100%)	4 (100%)
Do you have or have you had COVID-19?							
Yes	3 (7.7%)	1 (14.3%)		1 (12.5%)		1 (11.1%)	
No	33 (84.6%)	5 (71.4%)	3 (75.0%)	7 (87.5%)	7 (100%)	7 (77.8%)	4 (100%)
I don’t know	3 (7.7%)	1 (14.3%)	1 (25.0%)			1 (11.1%)	
Have you been tested for COVID-19?							
Yes	23 (59.0%)	4 (57.1%)	3 (75.0%)	3 (37.5%)	3 (42.9%)	6 (66.7%)	4 (100%)
No	14 (35.9%)	1 (14.3%)	1 (25.0%)	5 (62.5%)	4 (57.1%)	3 (33.3%)	
I don’t know	1 (2.6%)	1 (14.3%)					
If you have been tested, what was the result?							
Positive	1 (2.6%)	1 (14.3%)					
Negative	23 (59.0%)	3 (42.9%)	3 (75.0%)	3 (37.5%)	3 (42.9%)	7 (77.8%)	4 (100.0%)
I don’t know	5 (12.8%)	1 (14.3%)	1 (25.0%)		1 (14.3%)	2 (22.2%)	

* Same participant.

**Table 3:** Number of quotes within the themes categorized by race/ethnicity.

Themes	Bangladeshi	Burmese	Filipino	Korean	Arab (Yemeni)	Japanese
Perceptions about COVID vaccines	6	7	8	14	17	4
Willingness to receive COVID vaccine 1. concerns about vaccine safety/science is new	20 (3: vaccine safety)	8 (6-vaccine safety)	10 (3: vaccine safety)	13 (7: vaccine safety)	5 (vaccine safety)	8 (6: vaccine safety)
Barriers to COVID-19 vaccination 1. lack of access 2. confusion on eligibility 3. distrust of mass vaccination sites 4. fear of long-term side effects	14 (7: confusion on eligibility; 1: distrust of mass vaccination sites; 1: fear of long-term effects)	2	4 (1: lack of access)	5 (lack of access)	2 (fear of long-term effects)	10 (3: lack of access)
Enablers of COVID-19 vaccination	7	2	10	1	0	1
Sources of COVID-19 vaccination info	18	3	2	1	7	8
Strategies to improve COVID-19 vaccination uptake	16	1	0	3	2	3

## Data Availability

Access to the dataset is not possible due to ethical approval restrictions.

## Appendix A. Listening Session Script

**Eastern Michigan University REACH Listening Session (via Zoom)**

**Welcome and Introductions**

Hello, my name is _____, and I would like to thank you for joining us today for this listening session on COVID-19 vaccine attitudes and perceptions. Please take a moment to briefly tell us your name and the organization you represent. After introductions, I will turn things over to [FACILITATOR’S NAME] for a brief situational update.

(Introductions around the phone/computer.)

Thank you to everyone. We are so glad to have you here today.

Before we begin with this discussion, we would like to go over the informed consent. All of you should have filled out the informed consent form online prior to joining this session. If you have not, please fill it out and submit it before rejoining.

**Informed Consent Script**

Your participation in this listening session is voluntary, and there will be no individual benefit from your participation. There will not be any negative effects if you decide you do not want to participate.

Your responses will be written anonymously and reported as a whole with other people’s responses. No one will know how you responded in the final report. We would like to hear your honest opinions about the topics we discuss. There are no right or wrong answers to any of our questions. We encourage you to speak openly and honestly about your opinions and experiences.

You can choose not to respond to a question at any time. You can also leave the listening session at any time. If one of my questions is unclear, please stop me and I’ll ask it in a different way. All information collected from these sessions will be stored securely and kept confidential. None of the comments you make during today’s discussion will be linked with your name in any way. The discussion should take about 60 min. For more information about this project, contact Olivia Ford, oford1@emich.edu.

**Brief Situational Update**

Thank you all for being here today. As you all are aware, the COVID-19 vaccine is being rolled out across the country. Frontline healthcare providers and residents of long-term care facilities have been the first ones to get the vaccine, followed by other priority groups such as essential workers, seniors, and those with underlying conditions. As of April 1^st^, the vaccine is available for the general public. You/your organization is an important part of this community, and you may offer insights on what your community is thinking about when it comes to getting the COVID-19 vaccine. It is important for us to understand the different issues that may affect whether people in the community get vaccinated or not, and what we can do to ensure everyone accepts and has access to the vaccine.

With that, I would like to give each of you a chance to share your thoughts and insights with us. We have prepared several questions in advance, so I would like to share a few of them and allow each of you to respond. However, we are also happy to “go off script”, as needed, if other questions emerge.

**Main Discussion**
**A. General Introduction**
1. To start, it would be helpful to understand how COVID-19 has affected your community through the course of this pandemic. How do you think the introduction of the COVID-19 vaccine will affect your community?
2. What do you think about the COVID-19 vaccine?
**B. COVID-19 Vaccine Attitudes in the Community**
3. What do people in your community think about the vaccine? What are some things you have heard from your community about the vaccine?
4. Do you think most people in your community would be willing get to the vaccine once it becomes available? Why or why not?
**C. Barriers to and Enablers of COVID-19 Vaccination in the Community**
5. What are the main reasons people in your community would want to get the vaccine? Do people want life to go back to normal?
  6. What are the main reasons people in your community may not want the vaccine?
Probe on information, misinformation, attitudes toward vaccine, fear of side effects, trust in medical system/healthcare workers, fear of sharing personal data collected at vaccination distribution sites with public health and government officials.
7. There’s a lot of information about the vaccine right now. What have you heard about the COVID-19 vaccine from sources you trust? How about information from sources you don’t trust?
8. How easy do you think it would be for people in your community to get a COVID-19 vaccine if they wanted one?
9. Do you find there are issues to accessing the COVID-19 vaccine? Are there work conflicts or dynamics within households that cause conflict?
10. Are there any key barriers that people in your community are likely to face if they went out to get a COVID-19 vaccine?
**D. Strategies to Improve Vaccine Confidence in the Community**
11. How do you think your organization (non-government organization, faith-based organization, etc.) can contribute so more people can have confidence in and access to the vaccine?
12. How do you think our organization can play a role in creating demand for the vaccine in your community?
  Probe on messaging content (making sure it is culturally and linguistically appropriate), information sources, managing misinformation, other communication materials, access to vaccination provider sites (including having medical interpretation services available), any virtual events, or campaigns.
